# Efficacy of Sinus Tarsal Approach Compared With Conventional L-Shaped Lateral Approach in the Treatment of Calcaneal Fractures: A Meta-Analysis

**DOI:** 10.3389/fsurg.2020.602053

**Published:** 2021-01-15

**Authors:** Dongmei Ma, Lei Huang, Bin Liu, Zhigang Liu, Xin Xu, Jianfeng Liu, Tianyue Chu, Liming Pan

**Affiliations:** Department of Hand and Foot Surgery, The First Hospital of Jilin University, Changchun, China

**Keywords:** sinus tarsal approach, onventional L-shaped lateral approach, calcaneal fracture, meta-analysis, calcaneal fracture non-union

## Abstract

**Background:** This study aims to compare the efficacy of the sinus tarsal approach (STA) with that of the conventional L-shaped lateral approach (CLSLA) in the treatment of calcaneal fractures by meta-analysis.

**Methods:** PubMed, Embase, Web of Science, the Chinese National Knowledge Infrastructure, and China Wanfang database were searched to collect clinical randomized or non-randomized controlled trials of STA and CLSLA in the treatment of calcaneal fractures from January 2010 to May 2020. The data were analyzed by Stata 15.0 software.

**Results:** A total of 12 clinical trials were included, all of which were retrospective studies, including 961 patients. The results showed that when STA was compared with CLSLA, there was no difference in operation time with mean difference (MD) = −5.51 [95% confidence interval (CI): −12.57 to 1.55, *P* > 0.05], less bleeding during operation with MD = −18.49 (95% CI:−23.79 to −13.18), no difference in Böhler angle after an operation with MD = 0.78 (95% CI: −0.09 to 1.65) and in Gissane angle with MD = −0.07 (95% CI: −1.90 to 1.77), no difference in American Orthopedic Foot and Ankle Society score with MD = 2.16 (95% CI: −1.07 to 5.38), higher-excellent and better rate of Maryland food function with relative ratio = 1.12 (95% CI: 1.04 to 1.20), and lower of incidence of postoperative complications with relative ratio = 0.23 (95% CI: 0.14–0.37).

**Conclusion:** STA was more effective than CLSLA in the treatment of calcaneal fractures. Moreover, STA had advantages in less intraoperative bleeding, higher-excellent and better rate of Maryland foot function, lower incidence of postoperative complications, and higher safety.

## Introduction

The calcaneus is the largest tarsal bone of the foot, of which the shape is irregular. Most of the bones on the inside of the calcaneus are cancellous bones with uneven density, and a thin layer of cortical bone surrounds the outer layer. A calcaneal fracture is a common type of fracture in daily life, which accounts for ~2.6% of total body fracture and 61% of tarsal fracture. Among calcaneal fracture patients, 20–30% are complicated with calcaneocuboid joint injury or spinal fracture (1, 2). Generally speaking, most calcaneal fracture patients are caused by falling from high altitude, and the injury energy is often severe, which is common in young and middle-aged people (3). If a calcaneal fracture occurs, the injury spreads to the calcaneal articular surface, which is called an intra-articular calcaneal fracture. Approximately 75% of calcaneal fractures are intra-articular fractures (4). From the anatomical perspective, the structure of the calcaneus is relatively complex, so there are a variety of external manifestations, clinical classifications, and treatment methods of intra-articular calcaneal fracture injury. As the local soft tissue coverage of the calcaneus bone is relatively weak, an inappropriate treatment will result in more severe complications (4). Common complications caused by fractures include postoperative infection at the incision, necrosis of surrounding tissue, collapse and depression at the arch of the foot, difficult reduction of the articular surface, widening of the calcaneus, stiffness of joints, and traumatic arthritis, heel pain, deformity, and dysfunction. In severe cases, it will cause residual disability, and the disability rate can reach ~30% (5, 6). The primary purpose of the treatment of the calcaneal fracture is to treat an articular surface injury, restore flatness of the subtalar articular surface, renovate the biomechanical characteristics and external outline of the calcaneus to a normal state, and reduce postoperative complications, striving to achieve the best postoperative treatment effect (7).

Sanders made the primary classification of posterior calcaneal fractures in the 1990s. Based on the development of CT imaging, Sanders Classification, which is a classical one, divides intra-articular calcaneal fractures into four types. It is mainly used to reflect the severity degree of the subtalar articular surface of calcaneus injure (8). Based on it, it has important guiding significance for the selection of treatment methods and prognosis evaluation of calcaneal fracture (9). The common surgical approach is the conventional large “L”-shaped incision on the lateral side of the calcaneus. The advantage of this approach is that it can well-expose the anatomical structure of the fracture, firmly fix the fracture site after reduction, and directly observe the compression of the fracture. A bone graft may be given if necessary (10). Although significant clinical results can be obtained via traditional surgical methods, the blood supply is often poor, a calcaneal fracture is often associated with severe soft tissue injury, and various serious complications may occur after the operation (11, 12). Sinus tarsal approach (STA) combined with lateral small incision's treatment of intra-articular calcaneal fractures has become a common method for minimally invasive treatment and has been gradually recognized by increasingly more clinicians (13).

At present, there are many pieces of literature about the treatment of calcaneal fractures by STA and conventional L-shaped lateral approach (CLSLA) with different conclusions. Because of the limitation of most individual study sample size, there is a lack of objective evaluation of the advantages and disadvantages of STA. This article systematically reviewed published clinical randomized controlled trials (RCTs) or non-RCTs of STA and CLSLA in the treatment of calcaneal fractures to provide a reference for clinical application.

## Method

### Bibliography Retrieval

This meta-analysis was conducted under the statement of Preferred Reporting Items For Systematic Reviews (14). PubMed, Embase, Web of Science, China National Knowledge Infrastructure, and China Wanfang database were searched for clinical RCTs or non-RCTs of STA and CLSLA in the treatment of calcaneal fractures from January 2010 to May 2020. Meanwhile, manual examinations were conducted for the reference literature. For instance, if a test report was not detailed or there is a lack of information, we tried to contact the author by letter to obtain the information. The keywords were calcaneal fracture, open reduction, internal fixation, sinus tarsal approach, and conventional L-shaped lateral approach. There was no language restriction.

### Inclusion and Exclusion Criteria

Inclusion criteria were as follows: (1) Studies comparing STA and CLSLA in the treatment of calcaneal fractures were included; RCTs and non-RCTs were preferred to include RCT, whether blind or distributive hidden; (2) if the relevant RCT could not be found, non-RCT was involved; (3) the studies included clinically diagnosed calcaneal fracture and intra-articular fracture with joint displacement >2 mm; after CT and X-ray imaging examination, Sanders classification of type II or III was confirmed; (4) the studies were about fresh closed fracture; (5) the follow-up time was ≥6 months.

Exclusion criteria were as follows: (1) The pieces of literature were an unclear description of the intervention measures operation; (2) the pieces of literature were an incomplete description of sample size and related indicators; (3) the pieces of literature were repeated publication; (4) review, abstract, or other types of literature were excluded.

### Intervention Measures

The observation group was treated with STA, whereas the control group was treated with CLSLA.

### Document Quality Evaluation

Two evaluators conducted a bias risk assessment according to the Cochrane 5.1 bias risk assessment criteria (15). The independent evaluation included in clinical studies and inconsistencies were agreed upon through the intervention of a third evaluator after discussion. The following aspects were evaluated: (1) the generation of the random allocation scheme; (2) the concealment of the distribution scheme; (3) the implementation of the blind method; (4) the integrity of the resulting data; (5) the non-selective reporting of results; (6) other sources of bias. Low risk suggested low bias risk, high risk showed high bias risk, and unclear risk indicated that the literature did not provide sufficient or uncertain information on bias assessment.

### Data Extraction

Data extraction was carried out after the full text was read through, which was completed independently by two evaluators. In case of disputes, a third evaluator was involved in the discussion. The basic extracted information included the selection standard and sample size of the sample, the method and process of sampling and grouping, the basic data of the study object, the condition of the study, the content of the intervention, the measurement index, the duration of follow-up, the case turnover rate, and the cause of loss. The extracted outcome indexes included operation time, intraoperative blood loss, postoperative Bohler angle, postoperative Gissane angle, postoperative complications, American Orthopedic Foot and Ankle Society (AOFAS) score at the last follow-up, and the excellent and good rate of Maryland foot function score at the last follow-up.

### Statistical Processing

Stata 15.0 software was used for data analysis, and Revman 5.3 software was used for risk assessment. The *I*^2^ test estimated heterogeneity among studies. If *P* > 0.05 and *I*^2^ < 50%, it showed that heterogeneity among studies was not significant, then the fixed-effects model (FEM) was used for data analysis; If *P* ≤ 0.05 or *I*^2^ ≥ 50%, it indicated there was extensive heterogeneity among studies, and the random-effects model (REM) was used for combined analysis. Relative risk (RR) was used as the effective index for counting data, and mean difference (MD) was used as the effective index for continuous data. Meanwhile, the funnel plot and Egger's test were used to assess publication bias. The sensitivity of each index was analyzed to evaluate the robustness of the results.

## Results

### Basic Characteristics and Quality Evaluation of Included Studies

**Figure 1:**
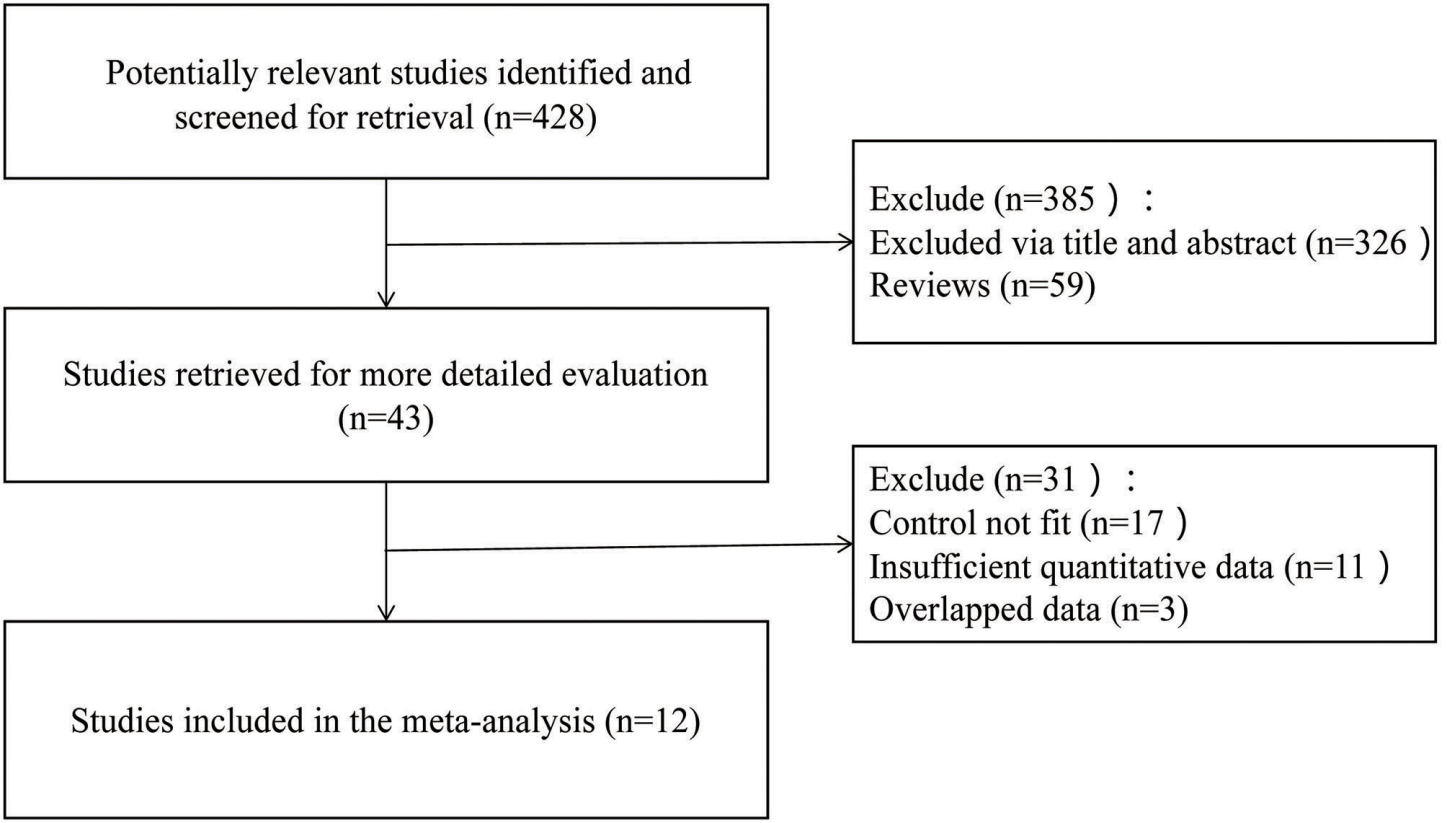
Flow diagram of literature screening.

A total of 428 potential articles were yielded in our preliminary literature search, and 43 articles were initially included after the titles and abstracts were screened. Twelve qualified articles (16–27) with a total of 961 subjects were enrolled eventually after the full-text manuscripts were further assessed. The selected studies were all retrospective researches. The general situation and baseline characteristics of the included studies are shown in Table 1. The literature screening process is shown in Figure 1.

**Table 1:** General situation and baseline characteristics of the included study.

**First author**	**Year**	**Country**	**Age (years old)**		**Fracture cases**		**Follow-up (month)**		**Outcomes**
			**STA**	**CLSLA**	**STA**	**CLSLA**	**STA**	**CLSLA**	
Shi et al.	2013	China	22~54	25~56	15	15	12~20	15~24	②③④⑥
Xia et al.	2014	China	20~67	19~67	59	49	8~28	8~28	①⑥
Liu et al.	2015	China	31.1 ± 6.1	33.1 ± 4.5	18	20	14.15 ± 2.68	15.01 ± 3.42	①②④⑤⑥
Basile et al.	2016	Italy	41.9 ± 11.6	39.6 ± 13.2	18	20	24	24	②③④
Huang et al.	2017	China	35.3 ± 6.9	35.8 ± 7.2	33	35	12	12	①②④⑤⑥
Scheper et al.	2017	Netherlands	37~59	39~56	65	60	12~28	12~28	②
Jian et al.	2019	China	37.2 ± 8.5	37.1 ± 8.4	40	40	12	12	①②④⑤⑥
Xia et al.	2019	China	44.5 ± 8.1	43.6 ± 8.3	43	43	12	12	②④⑥
Chen et al.	2019	China	38.6 ± 10.4	42.4 ± 11.8	45	45	6	6	②③④⑤⑥
He et al.	2019	China	39.4 ± 9.8	39.3 ± 9.5	78	78	24~81	24~81	①②④⑤
Shao et al.	2019	China	36.3 ± 8.1	35.3 ± 8.5	42	42	6	6	④⑤⑥
Wang et al.	2019	China	43.51 ± 8.77	42.84 ± 9.62	23	23	24	24	②④⑤⑥

*①Excellent and good rate of Maryland; ②Complication; ③AOFAS score; ④Operation time; ⑤Intraoperative bleeding volume; ⑥Böhler angle; ⑦Gissane angle; STA, Sinus Tarsal Approach; CLSLA, Conventional L-shaped Lateral Approach; AOFAS, American Orthopedic Foot & Ankle Society*.

### Bias Risk Assessment of Included Studies

**Figure 2:**
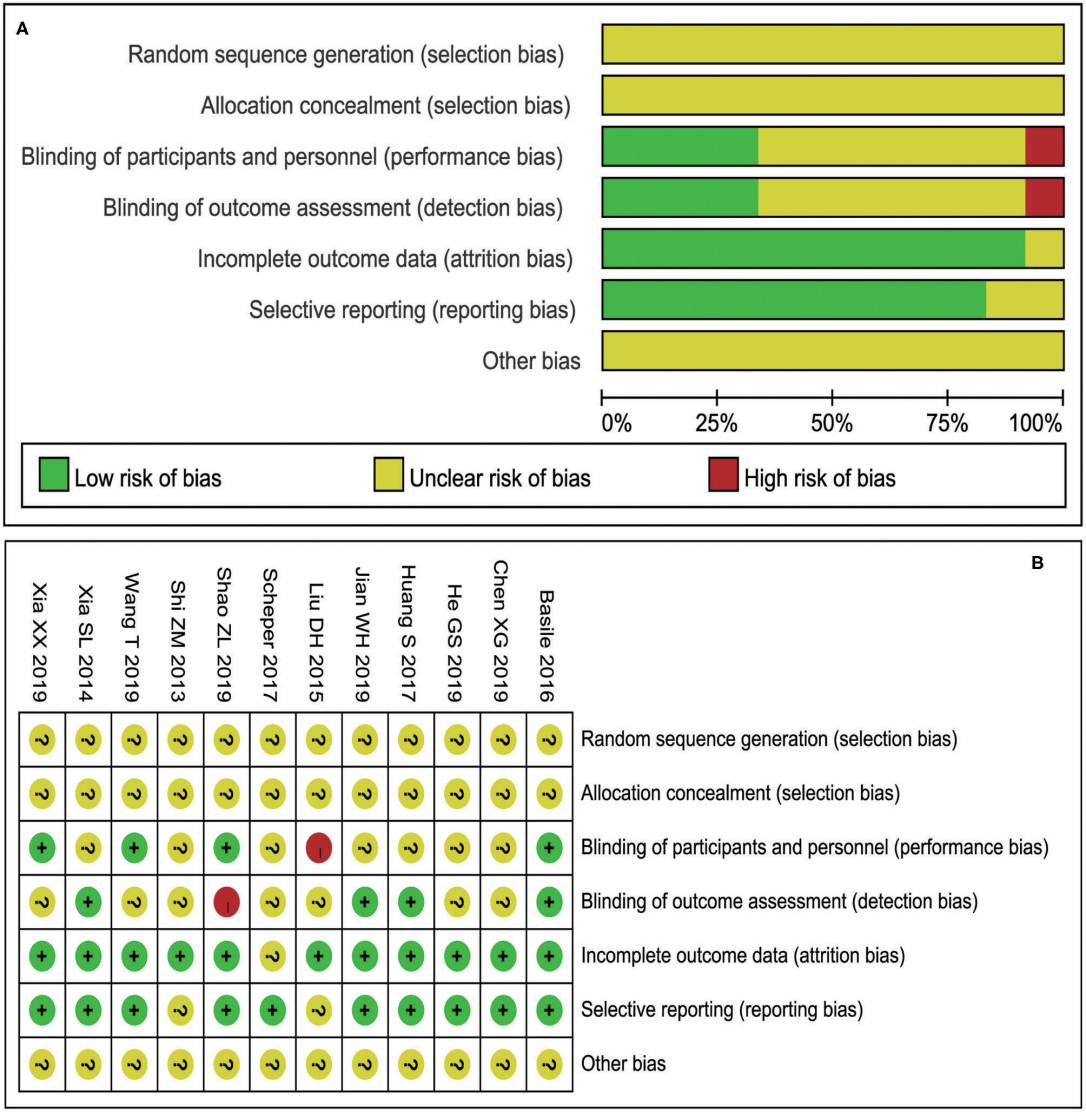
Risk assessment results included in the study (**A**: Risk of bias graph; **B**: Risk of bias summary).

According to the bias risk assessment method recommended by the Cochrane collaboration network, 12 research baselines included were comparable, all of which nevertheless had different levels of bias (Figures 2A,B). All the studies did not describe in detail whether the research sequence was randomly generated and assigned or not. Therefore, the risk assessment of random projects is not clear.

### Meta-Analysis Results

#### Excellent and Good Rate of Maryland Foot Function

**Figure 3:**
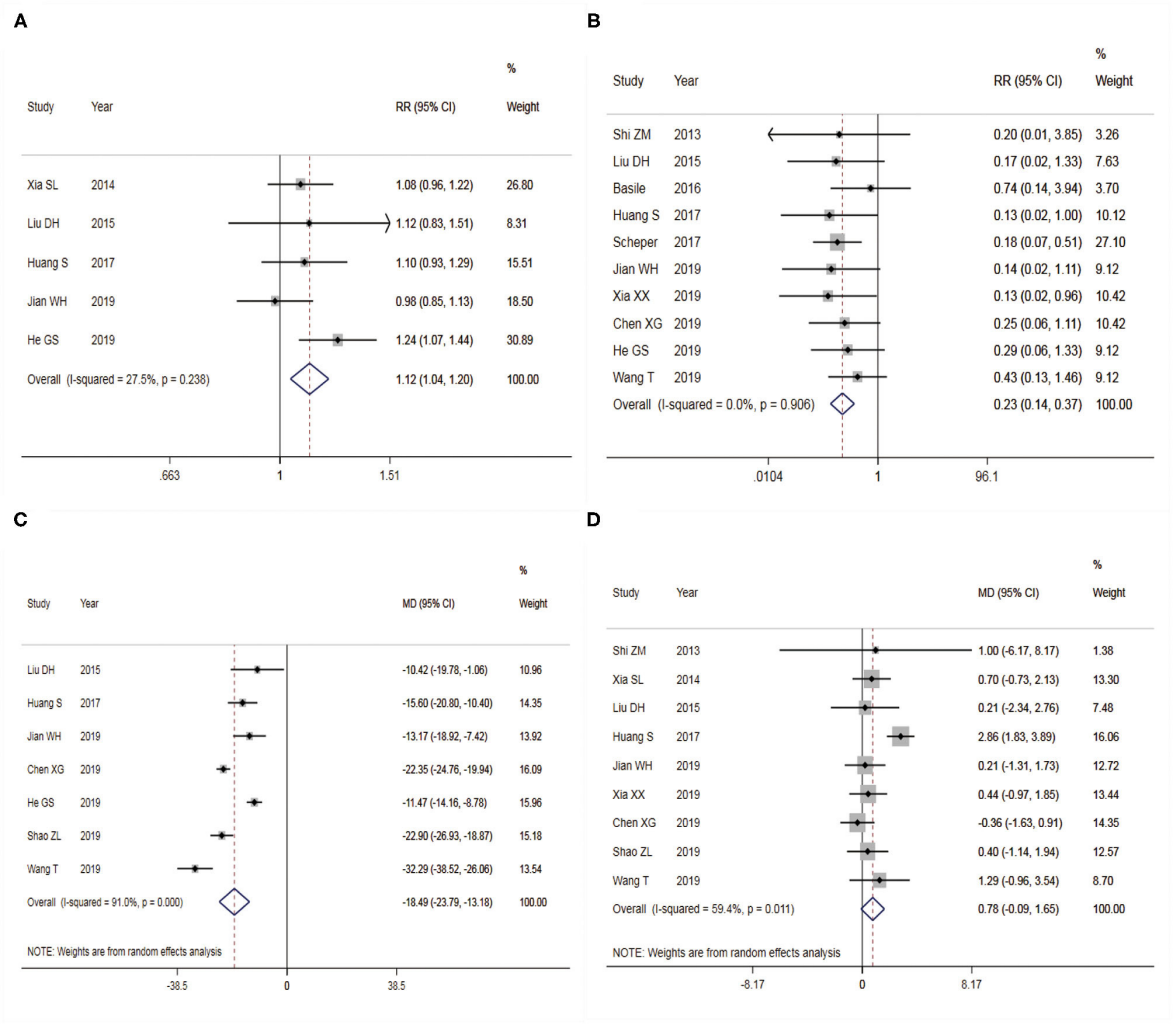
Forest plots of two surgical procedures with associated outcome indicators (**A**: excellent and good rate of Maryland foot function; **B**: postoperative complications; **C**: intraoperative blood loss; **D**: Böhler angle). Left side of the X-axis favors the Sinus tarsal approach; right side of the X-axis favors the conventional L-shaped lateral approach. RR, relative ratio; MD, mean difference. Red dotted line shows the pooled effect size.

A total of five studies were included in the analysis of the excellent and good rate of foot function in postoperative Maryland, with 458 patients enrolled. There was no heterogeneity among studies (*I*^2^ = 27.5%), so a FEM was used for combined analysis. The results showed that there was a significant difference in excellent and good rate of Maryland foot function after calcaneal fracture operation between the two different surgical approaches (Figure 3A), RR = 1.12 [95% confidence interval (CI): 1.04–1.20, *P* < 0.05]. It showed that the excellent and good rate of Maryland foot function after treatment of calcaneal fracture by STA was higher than that by CLSLA. The *P*-value of Egger's test was more than 0.05, indicating that there was no publication bias.

### Post-operative Complication

**Figure 4:**
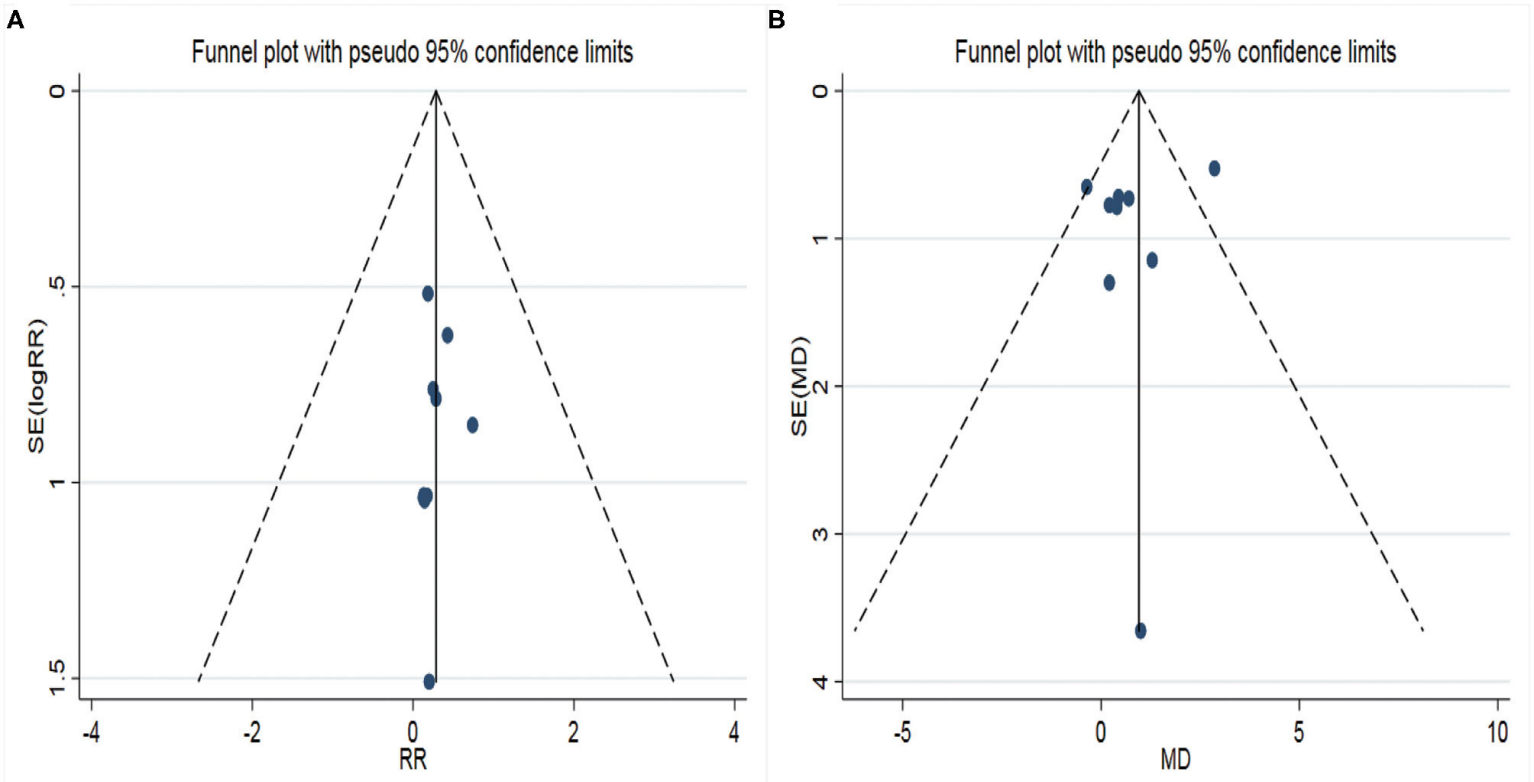
Funnel plots of two surgical procedures with associated outcome indicators (**A**: postoperative complications; **B**: Böhler angle). RR, relative ratio; SE, standard error; MD, mean difference. X-axis represents relative ratio (RR); Y-axis represents the standard error of log RR.

A total of 10 studies were included in the analysis of postoperative complications, containing 760 patients. There was no heterogeneity among studies (*I*^2^ = 0.0%), so FEM was adopted for analysis. The results showed a significant difference in the postoperative complication between the two different surgical approaches to treat calcaneal fracture (Figure 3B), RR = 0.23 (95% CI: 0.14 to 0.37, *P* < 0.05). It showed that the complication of STA was lower than that of CLSLA in the treatment of calcaneal fractures. The funnel plot (Figure 4A) was basically symmetrical, and the *P*-value of Egger's test was more than 0.05, indicating no publication bias.

### Amount of Intraoperative Bleeding

Seven studies were included to analyze the amount of intraoperative blood loss, consisting of 565 patients. There was heterogeneity across studies (*I*^2^ = 91.0%), so REM was chosen to analyze the data. The results showed statistical significance in the different bleeding amounts between the two surgical approaches during the fracture operation of treating calcaneal fracture (Figure 3C). When STA was compared with CLSLA, the results showed that MD = −18.49 (95% CI:−23.79 to −13.18, *P* < 0.05). The *P*-value of Egger's test was more than 0.05, indicating that there was no publication bias.

### Böhler Angle

Nine studies were incorporated in the analysis of the postoperative Böhler angle, including 642 patients. There was high heterogeneity among the studies (*I*^2^ = 59.4%), so we used a REM to combine the data. The results showed statistically significant differences in the post-operation Böhler Angle between the two surgical approaches to treat calcaneal fracture (Figure 3D). When STA was compared with CLSLA, the results showed MD = 0.78 (95% CI:−0.09 to 1.65, *P* > 0.05). The symmetry of the funnel plot was general (Figure 4B), whereas the *p*-value of Egger's test was more than 0.05, indicating there was a certain publication bias.

### Other Indicators

The comparison of the two surgical methods in AOFAS score, Gissane angle, and operation time is shown in Table 2. The results showed that the difference was not statistically significant.

**Table 2:** Main results of meta-analysis.

**Index**	***n***	**RR**	**MD**	**95% CI**	***P***	***I*^**2**^ (%)**	***p* for Heterogeneity**	**Model**	***P* for Publication bias (Egger)**
Excellent and good rate of Maryland	5	1.12	NA	1.04~1.20	0.002	27.5	0.238	FEM	0.876
Complication	10	0.23	–	0.14~0.37	0.000	0.0	0.906	FEM	0.735
AOFAS score	3	NA	2.16	−1.07~5.38	0.191	0.0	0.425	FEM	0.604
Operation time	10	NA	−5.51	−12.57~1.55	0.127	96.8	0.000	REM	0.157
Intraoperative bleeding volume	7	NA	−18.49	−23.79~-13.18	0.000	91	0.000	REM	0.965
Böhler angle	9	NA	0.78	−0.09~1.65	0.080	59.4	0.011	REM	0.443
Gissane angle	9	NA	−0.07	−1.90~1.77	0.943	61.7	0.008	REM	0.473

*n, number of study; NA, not applicable; RR, relative risk; MD, mean difference; FEM, fixed effect model; REM, random effect model; AOFAS, American Orthopedic Foot & Ankle Society*.

### Sensitivity Analysis

**Figure 5:**
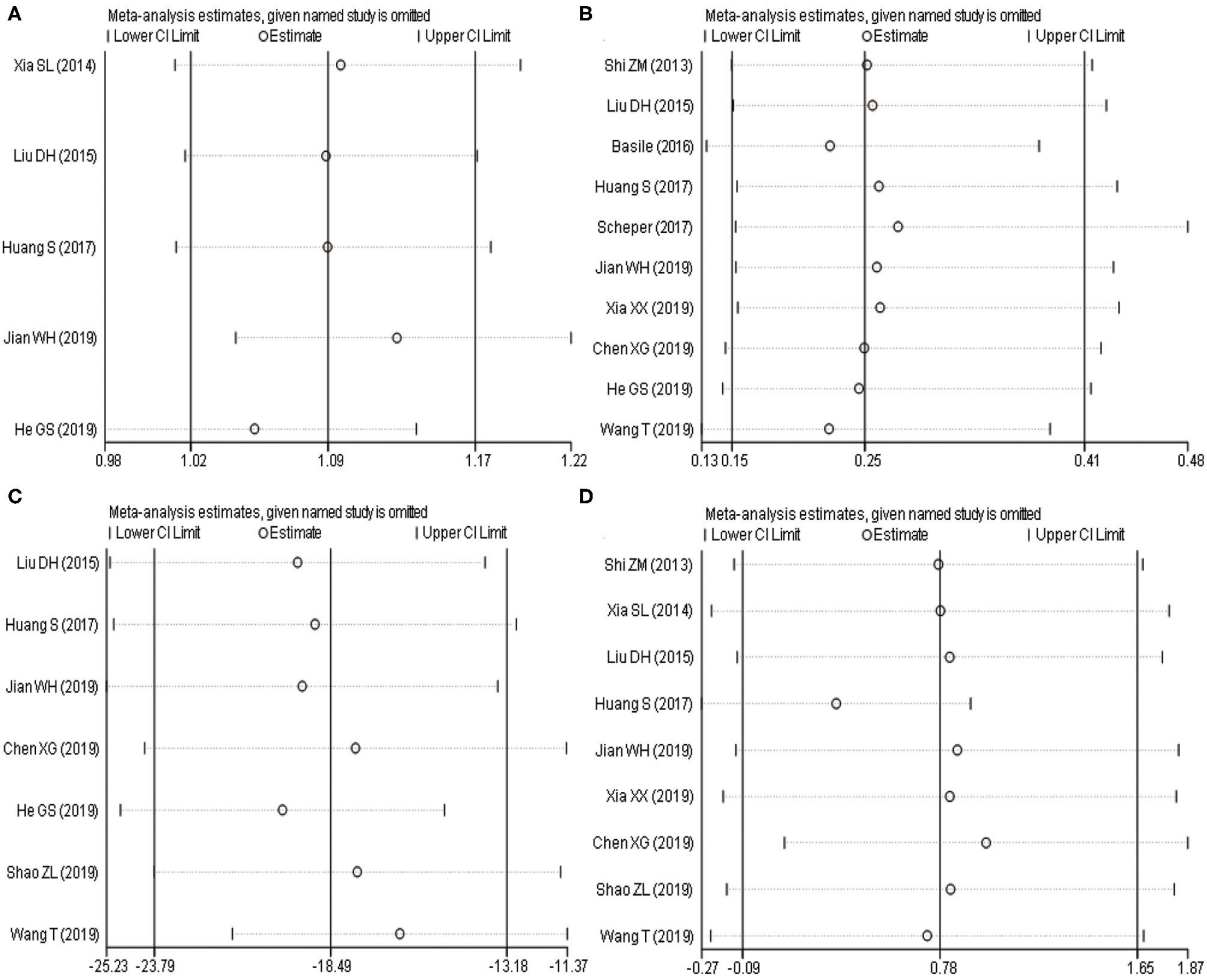
Sensitivity analysis of two surgical procedures with associated outcome indicators (**A**: excellent and good rate of Maryland foot function; **B**: postoperative complications; **C**: intraoperative blood loss; **D**: Böhler angle). **(A,B)** Horizontal line shows relative ratio; **(C,D)** Horizontal line shows mean difference.

The results (Figures 5A–D) of sensitivity analysis of the excellent and good rate of Maryland foot function, postoperative complications, intraoperative blood loss, and postoperative Böhler angle showed that there was no statistically significant change in postoperative complications and intraoperative blood loss when single literature was excluded and meta-analysis was performed. When one article was excluded from the analysis of the excellent and good rate of Maryland foot function, the difference was not statistically significant. On the other hand, when one article was excluded from the analysis of the Böhler angle after the operation, the difference was statistically significant. It showed that a cautious conclusion should be drawn when comparing the differences of Böhler angle and excellent and good rate of Maryland foot function between the two surgical methods.

## Discussion

Complex calcaneal fractures comminuted under the subtalar articular surface can be effectively exposed and reduced through a lateral extended L incision, of which the entry can well-expose the comminuted lateral wall of the calcaneus, posterior talus articular surface, tarsal sinus, and calcaneocuboid joint. However, this kind of incision requires that the whole skin contain subcutaneous soft tissue and fascia be lifted from the lateral wall of the calcaneus; furthermore, the long and short fibula muscle and its sheath should be pulled apart. During the operation, the sural nerve and the lateral calcaneal artery (28) need to be avoided to protect the unique hairless skin on the heel to prevent soft tissue complications (29). Even if the L incision is enlarged from outside carefully, skin necrosis at the edge of the incision, soft tissue and bone infection, and joint fibrosis and stiffness of the subtalar joint cannot be eliminated. The tarsal sinus incision starts from the tip of the lateral malleolus parallel to the long and short fibula muscle and passes through the tarsal sinus to the calcaneocuboid joint with an arc. Compared with the lateral extended L incision, the tarsal sinus incision requires less soft tissue to be moved. When the calcaneal fracture cannot be recovered percutaneously, and the operator needs to recover the calcaneal talus joint under direct vision, the STA shows its advantage (30). When STA is applied, the long and short fibula muscle and its sheath need to be pulled in the plantar flexion position so that the subtalar joint can be seen directly from the incision mentioned earlier. The larger extra-articular fracture can be recovered via percutaneous prying, and the subtalar intra-articular bone can be recovered under direct vision. After recovery, percutaneous screw fixation is used directly (31), or a plate is placed below the fibula tendon with screw fixation (32). The results of this study showed that the incidence of postoperative complications of STA was lower than that of CLSLA in the treatment of calcaneal fractures, which was verified by sensitivity analysis. It can be considered that STA is superior to CLSLA in terms of surgical safety. When applying the STA, there is no need to cut off the calcaneus-fibular ligament and the retinaculum under the fibular muscle; meanwhile, the joint space can be increased by proper turn inside during the operation. The STA has the advantages of simple operation, small incision, less soft tissue injury, low requirement for soft tissue, early operation, less periosteal peeling, little influence on the blood circulation of the fractured mass, and effectively reducing the occurrence of postoperative complications; meanwhile, it can achieve the effect of minimal invasion (33). Naturally, STA has higher requirements for surgeons who need a longer learning cycle. Also, sural nerve injury is considered to be the most common complication for the STA (34). Zhang et al. (35), in a meta-analysis including eight studies, showed that the complications of STA are significantly lower than those of CLSLA for calcaneal fractures. It is consistent with the results of this study.

In terms of surgical effect, there was no difference in operation time, postoperative Gissane angle, postoperative Böhler angle, and last AOFAS score between STA and CLSLA for calcaneal fracture. However, there was a significant difference in intraoperative blood loss and an excellent and good rate of Maryland foot function between the two approaches. STA has less bleeding and a higher-excellent and good rate of Maryland foot function. It is considered that STA should be recommended for patients with calcaneal fractures of type Sanders II and III, no obvious medial displacement of the calcaneus, and no obvious severe comminution of the calcaneal body. Nevertheless, there was a certain heterogeneity among the studies, which would affect the accuracy of the results to a certain extent. The causes of heterogeneity were related to the operator's surgical proficiency, postoperative nursing, etc.

Inevitably, the study also has some limitations: (1) As it was difficult to implement completely randomized and blind methods in orthopedic surgery, this study also included clinical non-RCTs. Besides, the original literature could not determine whether they were randomized or not. Hence, the quality of the articles included needs to be improved. (2) Most of the subjects included in this study were Chinese people, and there was bias in some outcome indicators. Although extensive retrieval strategies were adopted, the potential publication bias could not be exterminated. (3) There was heterogeneity in the operation time, intraoperative blood loss, and postoperative Böhler angle. (4) The sensitivity analysis of the postoperative Böhler angle and excellent and good rate of Maryland showed that the results were not robust, indicating that the conclusions about the indexes should be cautious.

Although STA has some shortcomings and complications in the clinical treatment of calcaneal fractures, compared with CLSLA, it has the advantages of less intraoperative bleeding, less postoperative complications, higher-excellent and good rate of Maryland foot function, and better curative effect. This difference is closely related to the anatomical characteristics of the two approaches, the exposure of the operation, and the skill level of the operation. It is suggested that STA is more recommended for patients with fracture displacement and mild comminution. The conclusions mentioned earlier have indispensable guiding significance for the current treatment of a calcaneal fracture. However, considering that there are still some limitations in this study, the conclusion still requires larger samples, researches of higher quality, and the use of critical indicators. Clinically, a reasonable surgical plan should be chosen according to the condition of patients and the severity of the fracture to further demonstrate its curative effect.

## Data Availability Statement

The original contributions presented in the study are included in the article/supplementary materials, further inquiries can be directed to the corresponding author/s.

## Author Contributions

DM, LH, and LP contributed to the study conception and design. All authors collected the data and performed the data analysis, contributed to the interpretation of the data, the completion of figures and tables, contributed to the drafting of the article, and final approval of the submitted version.

## Conflict of Interest

The authors declare that the research was conducted in the absence of any commercial or financial relationships that could be construed as a potential conflict of interest.
